# Molecular Control of Redox Homoeostasis in Specifying the Cell Identity of Tapetal and Microsporocyte Cells in Rice

**DOI:** 10.1186/s12284-019-0300-3

**Published:** 2019-06-18

**Authors:** Jing Yu, Dabing Zhang

**Affiliations:** 1 Joint International Research Laboratory of Metabolic & Developmental Sciences, Shanghai Jiao Tong University-University of Adelaide Joint Centre for Agriculture and Health, School of Life Sciences and Biotechnology, Shanghai Jiao Tong University, 800 Dongchuan Rd, Shanghai, 200240 People’s Republic of China; 2 School of Agriculture, Food and Wine, University of Adelaide, Waite Campus, Urrbrae, SA 5064 Australia

**Keywords:** Redox homoeostasis, Anther cell specification, Tapetal PCD, Rice

## Abstract

In flowering plants, male reproduction occurs within the male organ anther with a series of complex biological events including de novo specification of germinal cells and somatic cells, male meiosis, and pollen development and maturation. Particularly, unlike other tissue, anther lacks a meristem, therefore, both germinal and somatic cell types are derived from floral stem cells within anther lobes. Here, we review the molecular mechanism specifying the identity of somatic cells and reproductive microsporocytes by redox homoeostasis during rice anther development. Factors such as glutaredoxins (GRXs), TGA transcription factors, receptor-like protein kinase signaling pathway, and glutamyl-tRNA synthetase maintaining the redox status are discussed. We also conceive the conserved and divergent aspect of cell identity specification of anther cells in plants via changing redox status.

As other flowering plants, rice (*Oryza sativa*) male gametophyte development occurs within the male organ, stamen which contains anther and the supporting filament. At the early stages from stage 1 to stage 7 referring to staging by Zhang et al., (2011), the most critical events are the cell division and cell fate specification for the formation of somatic anther wall cell layers and reproductive cells, microspore mother cells (MMC), also called as pollen mother cells (PMC). At stage 1, three cell layers called as Layer 1 (L1), L2 and L3 are formed from an anther primordium. L2 cells perform cell division and form two layers of L2-derived (L2-d) cells while L1 differentiates into epidermis at stage 2. The outer L2-d differentiates into primary parietal cells (PPC), while the inner one differentiates into archesporial (Ar) cells at stage 3. PPC undergoes an asymmetric cell division and produces the endothecium and secondary parietal cells (SPC), while Ar cells differentiate into sporogenous cells (Sp) at stage 4. SPC undergoes symmetric cell division and cell differentiation into middle layer and tapetum at stage 5. Till stage 5, the Sp cells localize at the center of each anther lobe surrounded by four anther somatic wall layers: from the surface to interior, the epidermis, endothecium, middle layer, and tapetum. Then the meiosis of MMC occurs at stage 7 (Zhang et al., 2011; Zhang and Yang 2014). The tapetum, the innermost of the four sporophytic layers of the anther wall directly contacts with the developing gametophytes and acts as a nutritional source for the development of microspore by undergoing degeneration triggered by programmed cell death (PCD) from stage 7 to stage 11. At stage 8, after two rounds of cell division during meiosis, the tetrad covered by callose is formed. At stage 9, the haploid microspore is released into the lobe after callose degradation, followed by undergoing mitosis and pollen wall development and maturation with the accumulation of starch and lipids (Fig. 1) (Zhang et al., 2011; Ariizumi and Toriyama, 2011). As the advance of functional genomics, genetic and biochemical approaches, various components such as transcription factors, enzymes for lipids synthesis, transporters, kinases etc. involved in the anther development and pollen formation in rice have been reviewed (Shi et al., 2015; Zhao et al., 2016; Zhang and Yang, 2014; Cai and Zhang, 2018). This review paper focuses on the mechanism controlling the redox status regulation and its role in specifying anther cell identity and cell degeneration.

**Fig. 1:**
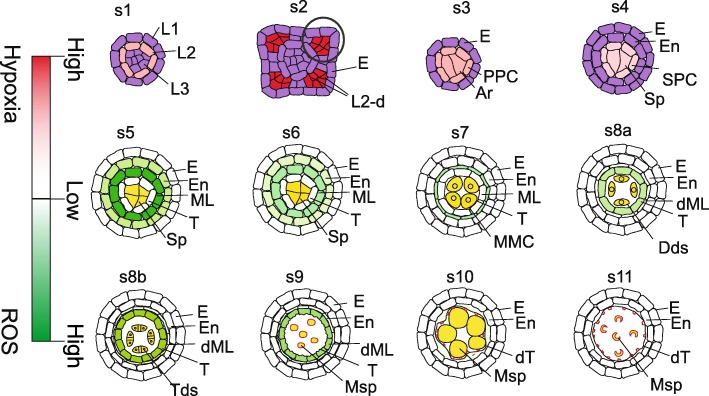
Redox status during anther development. Red color shows the gradient of hypoxia status; green color shows the gradient of ROS status; white color shows undetectable level of ROS by DAB staining; purple color shows no discussion about the redox status; yellow color shows the germinal cells

## Dynamics of Redox Status During Rice Anther Development

Reactive oxygen species (ROS) are produced in plants when molecular dioxygen (O_2_) is applied as a terminal electron acceptor, generating molecules such as hydrogen peroxide (H_2_O_2_), superoxide anion (O_2_-) and hydroperoxide radicals (OH^*^). Most of these ROS molecules are by-products of aerobic metabolism in plants (Miller and Mittler, 2006).

**Fig. 2:**
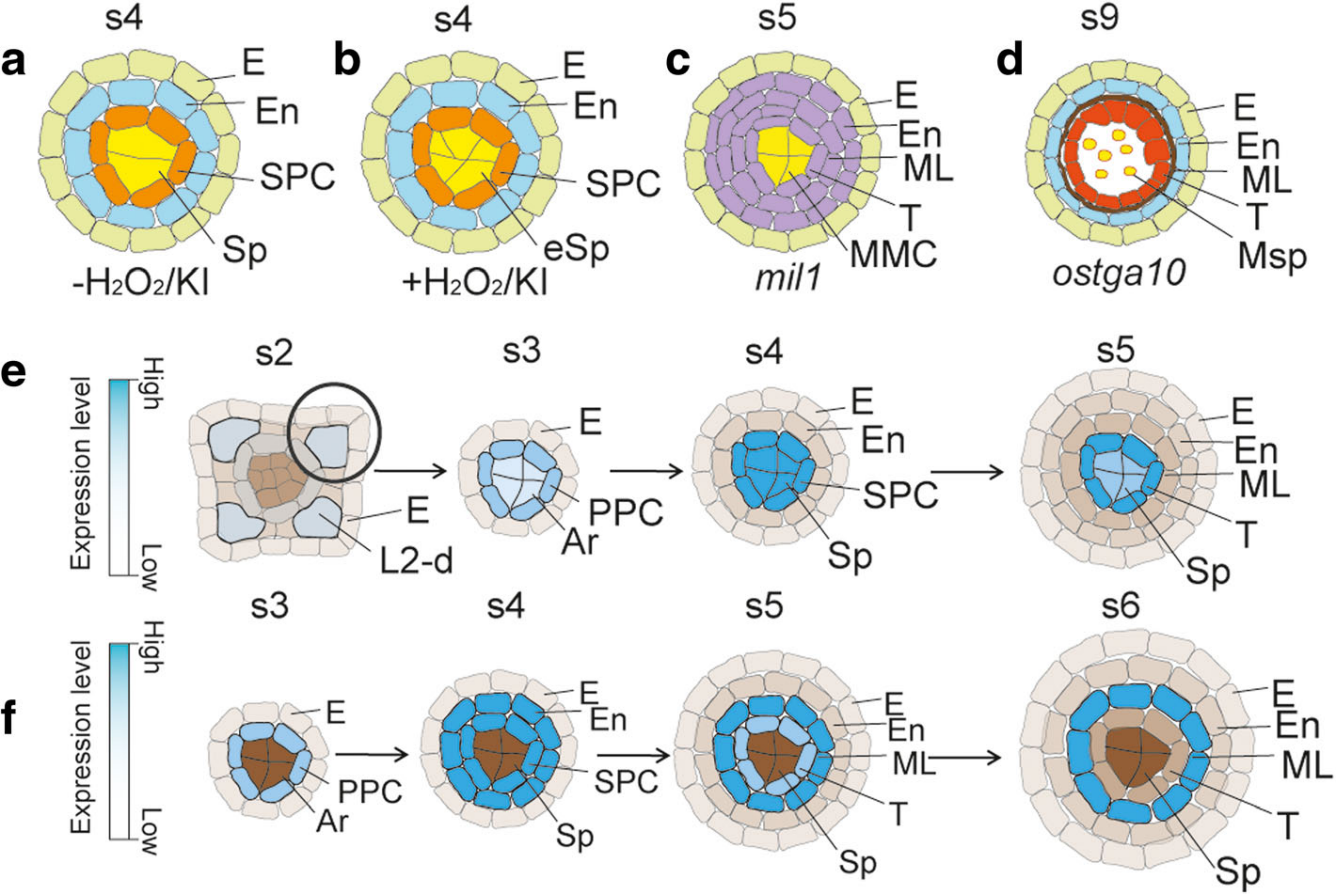
GRXs and TGAs are important for anther development. **a** Wild-type anther with normal number of Sp cells at stage 4, **b** Wild-type anthers treated with H_2_O_2_ or KI with excess Sp cells at stage 4, **c** Phenotype of *mil1* anther at stage 5, **d** Phenotype of *ostga10* anther at stage 9, **e** Expression pattern of *MIL1* in anther, **f** Expression pattern of *OsTGA10* in anther. Purple color in c shows abnormal somatic cell layers; blue color in e and f shows the gradient of gene expression; grey color in e and f shows different cell layers without the expression of *MIL1*(**e**) or *OsTGA10* (**f**)

In rice, ROS level is extremely low during anther cell specification stages, which is less than 200 pmol mg ^− 1^, and the ROS level increases twice at stage 4 to stage 5 (Yang et al., 2018). 3,3′-diaminobenzidine (DAB) staining indicates the accumulation of H_2_O_2_ in middle layer and endothecium from stage 5 to stage 9 and decrease of ROS at stage 10 and stage 11 (Fig. 1) (Hu et al., 2011; Yang et al., 2016; Yi et al., 2016; Yang et al., 2018). The increase of ROS in anther cell wall layers may play a key role in initiating promoting cell degeneration of middle layer and tapetum from the meiosis (Hu et al., 2011; Yi et al., 2016). These results also suggest that the low hypoxia status is critical for early anther cell specification (Yang et al., 2018). Supportively, during anther cell specification, individual treatment of H_2_O_2_ and the ROS-removal reagent potassium iodide (KI) treatment in wild-type anthers induces cell proliferation and increases the number of Ar cells and Sp cells (Fig. 2 a, b), highlighting the importance of redox homeostasis during anther cell specification in rice (Yang et al., 2018).

Consistent with the role of redox in specifying rice anther cell specification, the hypoxia status has been demonstrated in maize (*Zea mays*) (Kelliher and Walbot, 2012). In the airspace between the tassel and the innermost leaf in maize, O_2_ concentration is around 1.2 to 1.4% during anther cell specification, and 5 days after that, the O_2_ concentration reaches 5%. This result also highlights the transient hypoxia status during anther cell specification in maize, which is similar to that of rice (Kelliher and Walbot, 2012; Yang et al., 2018). The treatment of N_2_ of anther cells increases the cell numbers of Ars, while this induction is absent when treated with O_2_, indicating that low level of O_2_ induces the cell specification of Ars (Kelliher and Walbot, 2012). Furthermore, the defects of Ar cell specification of *msca1* (*male sterile converted anther 1*) with a mutation in a glutaredoxin (GRX) gene could be restored by reducing treatment (Sheridan et al., 1996, 1999; Kelliher and Walbot, 2012). These results suggest the importance of hypoxia status within growing anther tissue on stimulating the Ar cells development in maize (Kelliher and Walbot, 2012).

Although in *Arabidopsis*, the ROS level and redox status has not been measured during the anther development, the components controlling the redox status essential for male reproduction appeared to be conserved such as the the GRXs in *Arabidopsis* (Xing and Zachgo, 2010). Moreover, the mutation of an anther-expressed ROS synthetic gene *RBOHE* (*Respiratory Burst Oxidase Homologue E*) causes the defects of pollen development in *Arabidopsis* (Xie et al., 2014).

## Glutaredoxins (GRXs) and TGA Factors Control Redox Status During Early Rice Anther Development

**Fig. 3:**
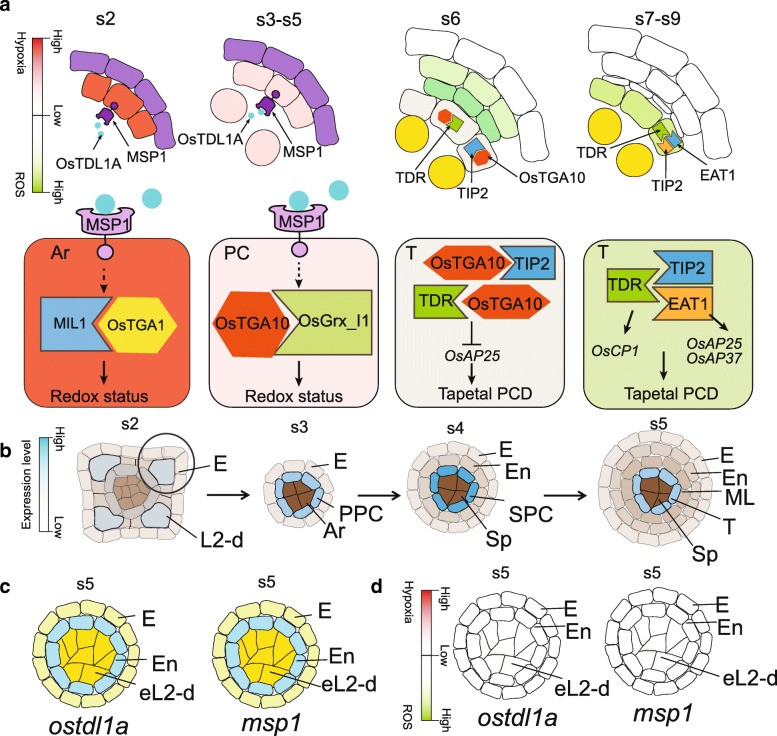
OsTDL1A-MSP1 pathway regulates anther development. **a** Model for protein interaction on tapetum development. **b** Expression pattern of *OsTDL1A* or *MSP1* in anther. **c** *ostdl1a* and *msp1* anthers with excess L2-d cells at stage 5. **d** *ostdl1a* and *msp1* anthers with undetectable ROS at stage 5. Red color in a shows the gradients of hypoxia status; green color in a shows the gradients of ROS level; purple color in a shows no discussion about the redox status; white color in a and d shows undetectable level of ROS by DAB staining; yellow color in a shows germ cells; blue color in b shows the gradients of gene expression; grey color in b shows different cell layers without the expression of *OsTDL1A* or *MSP1*

Glutaredoxins (GRXs) are small glutathione (GSH)-dependent oxidoreductases that catalyze the reversible reduction of protein disulfide bridges or protein-GSH mixed disulfide bonds, affecting various cellular events (Lemaire, 2004; Rouhier et al., 2004; Kelliher and Walbot, 2012; Zhang and Yang, 2014). Mutation in an anther-expressed glutaredoxin, *MIL1* (MICROSPORELESS1) in rice, fails in the differentiation of inner SPC into middle layer and tapetal cell layer (Fig. 2c). MIL1 belongs to CC-type glutaredoxin, which usually interacts with TGA (TGACGTCA cis-element-binding protein) proteins. MIL1 interacts with TGA1 (Hong et al., 2012b). While the biological function of TGA1 has not been reported yet. In rice, anther-expressed OsGRX_I1 has a physical interaction with OsTGA10, mutation of which exhibits defects in tapetal PCD (Fig. 2d) (Yang et al., 2016; Chen et al., 2017). The interaction between glutaredoxins and TGA proteins may cause the transcriptional activity changes of TGA proteins caused by Cys residue modification by glutaredoxin which remains to be investigated. *MIL1* expression is detectable in Ar cells from stage 2, extending to PPCs and Ar cells at stage 3 and SPCs as well as Sp cells at stage 4. At stage 5, *MIL1* is highly accumulated in tapetum and weakly detected in Sp (Fig. 2e), similar to *TGA1* (Hong et al., 2012b). However, no *OsTGA10* transcripts are detectable at stage 2. *OsTGA10* is detectable in PPC at stage 3 and the corresponding daughter cells endothecium and SPC at stage 4. At stage 5, *OsTGA10* is highly expressed in middle layer and weakly expressed in tapetum. Till stage 6, *OsTGA10* is specifically expressed in middle layer and has no expression during meiosis (Fig. 2f) (Yang et al., 2016). Overall, *MIL1* and *TGA1* expression pattern is slightly earlier and inner than that of *OsTGA10*. The differential expression of *TGA* genes may be associated with their verified function during anther development. Furthermore, *TGA* genes may have redundant function in specifying anther cells for the high expression pattern of *OsTGA11* and *OsTGA12* at premeiosis stages, partially overlapping with that of *OsTGA10* (Yang et al., 2016). Together with the evidence that OsTGA10 is required for suppressing the induced expression of a PCD executor gene *OsAP25* (*Oryza sativa Aspartic Protease 25*) by TIP2 (Niu et al., 2013; Chen et al., 2017), we hypothesize that TGA1-MIL1 and OsTGA10-OsGRX_I1 might play roles alternatively both spatially and temporarily, and the later might function as a link for anther cell specification and degeneration (Fig. 3a).

Evolutionarily, the function of GRXs on anther cell specification is conserved among rice, *Arabidopsis thaliana* and maize. The homologs of MIL1 include ROXY1 and ROXY2 in *Arabidopsis* (Xing and Zachgo, 2010), and MSCA1 (Male Sterile Converted Anther 1) in maize (Sheridan et al., 1996, 1999). *roxy1 roxy2* and *msca1* display defects in anther cell specification: adaxial lobes form *roxy1 roxy2* mutant fail to form Sp cell at stage 3, while the abaxial lobes develop normally till stage 5, with irregular PMC at stage 6; *msca1* mutant fails to form Ar cell at stage 3, indicating the conserved role of GRXs during anther development (Xing and Zachgo, 2010; Kelliher and Walbot, 2012; Yang et al., 2015). Furthermore, ROXY1 and ROXY2 interact with TGA9 and TGA10 during anther development, and *tga9 tga10* displays anther cell developmental defects similar with *roxy1 roxy2* (Murmu et al., 2010). MSCA1 interacts with TGA transcription factor FASCIATED EAR4 (FEA4) (Yang et al., 2015).

However, the MIL1-MSCA1-ROXY1/ROXY2 show divergent aspect during the anther development. The developmental defects of *msca1* anthers are seen as early as abortion of the differentiation of Ar cells from L2-d cells (Kelliher and Walbot, 2012). While *mil1* anthers have normal cell division and differentiation of Ar cells, forming the PPC and Sp cell as well as the initiation of the endothecium and epidermis at stage 5. However, the progenies of Sp cells of *mil1* anthers develop into many smaller cells and fail to enter meiosis (Hong et al., 2012b). These results indicate that the maize GRX mutant shows earlier developmental defects than that of rice. In addition, unlike the defects of *mil1* and *msca1*, *roxy1 roxy2* and *tga9 tga10* anthers display earlier abortion of adaxial lobes and later abortion of abaxial ones (Xing and Zachgo, 2010; Murmu et al., 2010). MSCA1 interacts with FEA4, controlling meristem size. Mutation in MSCA1, also named as *abph2*, displays large apical meristem, mimicking the phenotype of *fea4* (Pautler et al., 2015; Yang et al., 2015). However, one of the counterparts of ROXY1/ROXY2, PAN (PERIANTHIA), a homolog of FEA4, is not required for meristem size control in *Arabidopsis* (Li et al., 2009). Instead, *pan* mutant exhibits a pentamemrous flower and *roxy1* single mutant displays defects in petal initiation (Running, 1996; Li et al., 2009). The involvement of GRX-TGA counterparts in flower development and shoot apical meristem size control has not been reported in rice possibly due to that the divergent function of GRXs during evolution.

## OsTDL1A-MSP1 Signaling Pathway in Specifying Somatic and Reproductive Cell Identity

In rice anther, cell fate specification of germinal and somatic cells is also associated with cell surface-localized Leu-rich repeat receptor-like kinases (LRR-RLKs) and their putative ligands (Fig. 3a). *OsTDL1A* (*TPD1-like 1A*)/*MIL2*(*MICRO-SPORELESS 2*) encodes a small peptide with the expression in Ar cells at stage 3 and later radically in innermost somatic cell layer (Fig. 3b) (Hong et al., 2012a). *MSP1*(*MULTIPLE SPOROCYTE 1*) encodes a LRR-RLK expressed in the innermost somatic cell layer (Fig. 3b) (Nonomura et al., 2003). Both *ostdl1a* and *msp1* exhibit excessive Sp cells and lack of middle layer and tapetal cell layer (Fig. 3c). *ostdl1a msp1* double mutant displays similar phenotype with each single mutant (Yang et al., 2016). Furthermore, the 21-aa peptide of OsTDL1A is able to interact with MSP1, suggesting that OsTDL1A acts as a ligand of MSP1(Fig. 3a) (Nonomura et al., 2003; Hong et al., 2012a; Yang et al., 2016). Genetic and biochemical evidences show that OsTDL1A-MSP1 signaling specifies the early anther cell fate by stimulating the transition of parietal cells into the middle layer and tapetal cells, and suppressing the extra activity of generating microsporocytes in rice (Nonomura et al., 2003; Hong et al., 2012a; Yang et al., 2016). Genome-wide expression profiles also show the altered expression of genes in *ostdl1a* and *msp1–4* are associated with redox modulation such as ROS-producing and ROS-scavenging enzymes and proteins, including putative peroxidases, cytochrome P450s, oxidoreductases, thioredoxins, and glutaredoxins (Yang et al., 2016). Consistent with the expression alteration of genes involved in redox homeostasis, the presence of hydrogen peroxide in the middle layer and expression signal of glutaredoxins *OsGrx_I1* and *MIL1* in anther cells are detectable in rice anthers at stage 5 after the differentiation of the four cell wall layers, but not in *ostdl1a* and *msp1–4*. Furthermore, the ROS level is not detectable in *ostdl1a* and *msp1–4* anthers (Fig. 3d), highlighting the role of OsTDL1A-MSP1 signaling in specifying anther cell fate by directly or indirectly changing the redox status (Fig. 3a) (Yang et al., 2016). The OsTDL1A-MSP1 pathway is conserved in *Arabidopsis* and maize as reviewed by Zhang and Yang (2014), whether the counterparts of OsTDL1A-MSP1 play a role in regulating redox status during anther development remains to be elucidated.

## Glutamyl-tRNA Synthetase Determines Anther Cell Identity and Patterning Via Affecting Redox Status

**Fig. 4:**
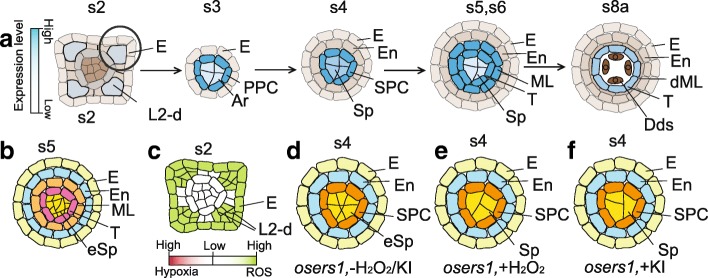
OsERS1 is required for redox regulation. **a** Expression pattern of *OsERS1* in anther, **b** *osers1* anther at stage 5 with excess Sp cells, **c** Higher ROS level in *osers1* anthers at stage 2, **d-f** *osers1* anthers at stage 4 treated with H_2_O_2_ or KI, **d** *osers1* anther without treatment, **e** *osers1* anther treated with H_2_O_2_, **f** *osers1* anther treated with KI. Blue color in a shows the gradients of gene expression; grey color in a shows different cell layers without *OsERS1* expression; green color in c shows the gradient of ROS level; white color in c shows no detectable redox status

Aminoacyl-tRNA synthetases (aaRSs) conjugate amino acid and their cognate tRNA, which is critical for protein synthesis and amino acid metabolism. aaRSs are widely distributed in the genome from all the species, including plants (Yamakawa and Hakata, 2010). *OsERS1* (*Oryza sativa Glutamyl-tRNA Synthetase)* is a housekeeping gene, however, *OsERS1* transcripts are detectable in cells of presumptive anther lobes at the four corners at stage 2, which is comparable with high hypoxia status in maize (Yang et al., 2018; Kelliher and Walbot, 2012). Later, the transcripts of *OsERS1* accumulate in PPC and Ar cells at stage 3, followed by in SPC and Sp cells at stage 4, and in the innermost somatic cell layer from stage 5 to stage 8 (Fig. 4a) (Yang et al., 2018). *osers1* anthers show over-proliferation and disorganization of L2-d cells, forming fused lobes and extra germ cells in early anthers (Fig. 4b). Biochemically, OsERS1 functions as a glutamyl-tRNA synthetase in ligating Glu with tRNA, regulating the amino acid metabolism and tricarboxylic acid cycle in anther. In *osers1* anthers, most of Glu family amino acids and the related metabolites including oxaloacetate and malate in tricarboxylic acid cycle, are over-accumulated (Yang et al., 2018). Global metabolomics profiling shows that *osers1* has the increased level of ROS produced by mitochondrial activities, and measurement also indicates hydrogen peroxide content in the *osers1* mutant at stages 4 to 5 is ∼900 pmol mg^− 1^, twice as that of wild-type anthers. Also, in the *osers1* mutant, the amount of hydrogen peroxide precursor, superoxide radical, is increased slightly. Consistently, DAB staining shows that *osers1* anthers display detectable H_2_O_2_ in anther primordium before stage 3 (Fig. 4c), and strong signals in anther cells at stages 4 to 5, which is earlier than that of the wild type (Yang et al., 2018). The application of 100 μM H_2_O_2_ and 1 mM potassium iodide (KI), the ROS-removal reagent cause the increased number of L2-d cells and Sp-like cells in wild-type anther, photocopying the defects of the *osers1* mutant (Fig. 2 a, b). But *osers1* anther treated with H_2_O_2_ and KI shows a decreased number of L2-d cells and Sp-like cells (Fig. 4 d-f) (Yang et al., 2018). Therefore, both the elevation of ROS by H_2_O_2_ injection and neutralization of ROS using KI disturb ROS homeostasis, which causes abnormal cell division, cell fate specification.

There are 45 aaRSs in *Arabidopsis*, however, only 21 aaRS mutants display developmental defects and chloroplast aaRS mutants exhibit an embryo defects, while mitochondrial aaRS mutants exhibit an ovule abortion phenotype with the exception of *ova9* showing defects in female transmission (Berg et al., 2005). None of the aaRS has been reported for the anther development in *Arabidopsis*. It might be contributed by the functional redundancy of aaRSs or the functional divergence of amino acid homeostasis on anther development.

## The Role of ROS in Promoting Tapetal PCD

Most of these ROS molecules are toxic in plants under abiotic stresses (Miller and Mittler, 2006). ROS also serves as important signaling molecules affecting a diverse range of plant processes, such as promoting programmed cell death (PCD) (Lam et al., 2001; Scherz-Shouval et al., 2007). Plants have gained diverse protective systems such as ROS-scavenging enzymes including superoxide dismutase, catalase, and peroxidase (Apel and Hirt, 2004), and non-enzymatic mechanisms, to modulate ROS levels (Mittler et al., 2004). The so-called non-enzyme system contains low molecular mass antioxidants including ascorbate, carotenoids, glutathione, and metallothioneins (MTs), which can remove hydroxyl radicals and singlet oxygen (Gechev et al., 2006).

**Fig. 5:**
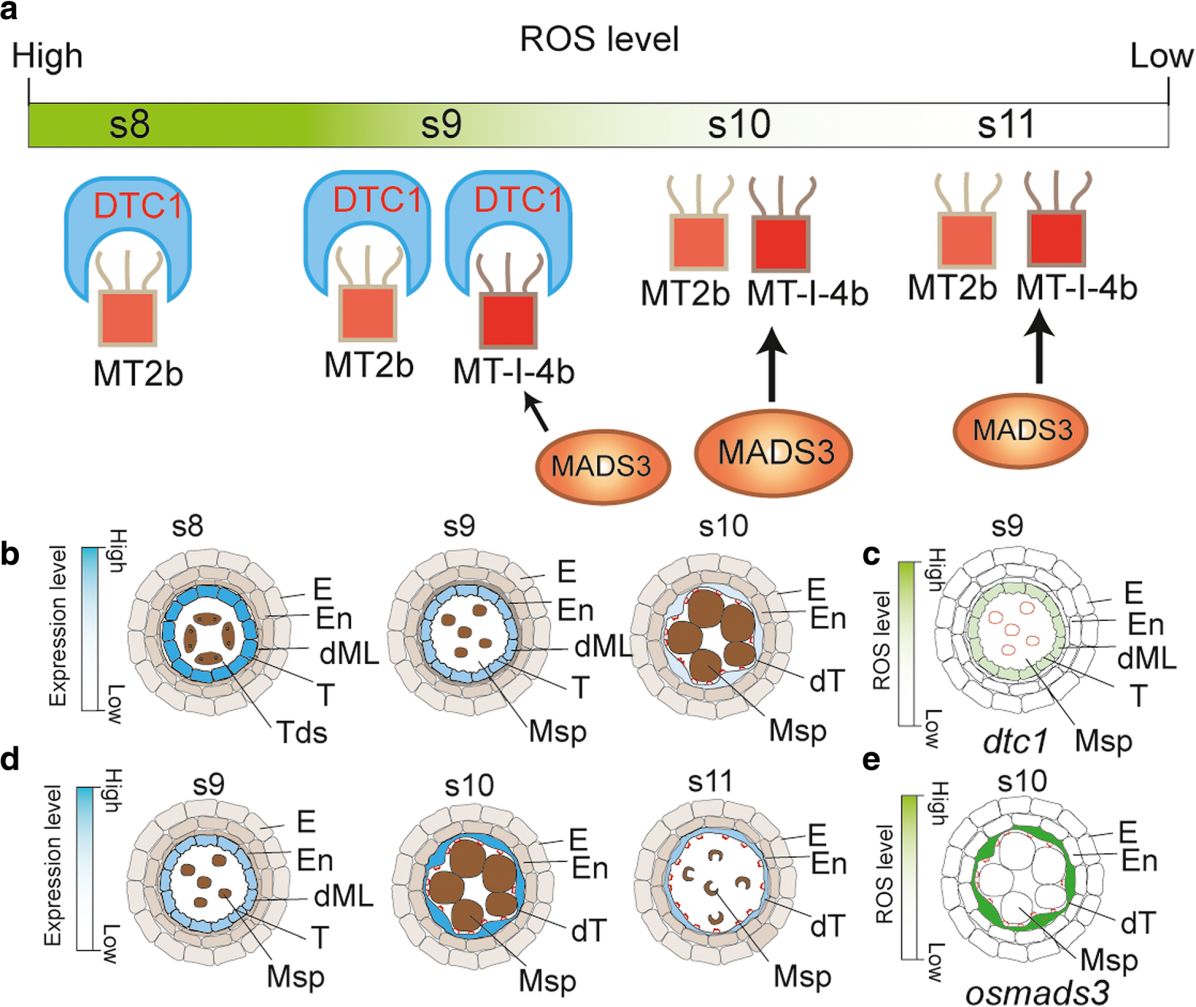
ROS scavengers and the regulatory network during tapetal PCD. **a** DTC1 and OsMADS3 control ROS level via regulating MT2b and MT-I-4b, **b** Expression pattern of *DTC1* in anther, **c** Phenotype of *dtc1* anther at stage 9, **d** Expression pattern of *OsMADS3* in anther, **e** Phenotype of *osmads3* anther at stage 10. Blue color in b and d shows the gradients of gene expression; grey color in b and d show s different cell layers without the expression of *DTC1* (**b**) and *OsMADS3* (**d**); green color in c and e shows the gradient of ROS level; white color in c and e shows undetectable level of ROS by DAB staining

Consistent with the role of ROS in promoting cell death, a high level of ROS is detectable from stage 7 during the meiosis when the tapetal cell death is initiated (Li et al., 2006; Hu et al., 2011; Niu et al., 2013; Fu et al., 2014). Some ROS molecules, such as the superoxide anion radical and hydrogen peroxide are key regulators of plant cell death (Overmyer et al., 2003; Gechev and Hille, 2005; Gadjev et al., 2008; Hu et al., 2011). Metallothioneins (MTs) belongs to low molecular mass antioxidants, with the ability to remove hydroxyl radicals and singlet oxygen (Gechev et al., 2006). OsMT2b and OsMT-I-4b are able to scavenge superoxide and hydroxyl radicals (Wong et al., 2004; Hu et al., 2011). At stage 8 and stage 9 during anther development, OsMT2b interacts with DTC1 (Defective Tapetum Cell Death 1), a KELCH repeat-containing protein, keeping the scavenging less effective, as a result, ROS level is high in the tapetal cells. After stage 9, as the expression level of *DTC1* decreases, the released OsMT2b thus reduces the ROS level (Fig. 5 a, b). Supportively, *dtc1* mutant displays low level of ROS and defective in PCD initiation (Fig. 5c) (Yi et al., 2016). Meanwhile, *OsMT-I-4b* is highly induced by a MADS box transcription factor, OsMADS3, highly expressed at stage 9- stage 11(Fig. 5d), leading to increase the ROS scavenge efficiency at stage 10 and stage 11. *osmads3* mutant displays higher level of ROS and abnormal tapetal PCD and pollen fertility (Fig. 5e) (Hu et al., 2011). These results indicate that the precise ROS level is of vital importance for tapetal PCD.

In addition, the redox regulator OsTGA10 interacts with two bHLH (basic Helix Loop Helix) transcription factor TIP2 (TDR Interacting Protein 2) and TDR (Tapetum Degeneration Retardation) (Li et al., 2006; Niu et al., 2013; Ji et al., 2013; Fu et al., 2014; Ko et al., 2014; Chen et al., 2017). The anther specific expressed bHLH transcription factors TIP2, TDR and EAT1 are tapetal PCD positive regulators functioning in a cascade manner. TIP2 interacts with TDR, and directly regulates the expression of *EAT1* (Fu et al., 2014; Ko et al., 2014). In addition, TIP2 regulates the expression of *TDR* directly (Fu et al., 2014). TDR interacts with EAT1, and TDR is required for the induction of *OsCP1* (*Oryza sativa Cysteine Protease 1*) (Li et al., 2006), while EAT1 is required for the induction of *OsAP25* (*Oryza sativa Aspartic Protease 25*) and *OsAP37* (Niu et al., 2013) (Fig. 3a). OsCP1, OsAP25 and OsAP37 are key executors of tapetal PCD in rice (Lee et al., 2004; Niu et al., 2013). In *Arabidopsis*, the homolog of TDR, AMS (ABORTED MICROSPORE), is a key regulator for tapetal PCD via regulating a series of PCD related genes (Xu et al., 2010; Xu et al., 2014), however, single mutants of TIP2 and EAT1 homologs, bHLH089, bHLH090, bHLH010 are fertile and *bhlh089 bhlh090 bhlh010* triple mutant displays tapetal PCD defects (Niu et al., 2013; Ji et al., 2013; Zhu et al., 2015), indicating the functional redundancy for the bHLHs on tapetal PCD in *Arabidopsis* and the conservation of the anther expressed bHLH family on tapetal PCD between rice and *Arabidopsis*.

## Conclusions

Plant male development is a coordinated process starting form an anther primordium, undergoing cell division and differentiation, meiosis, tapetal PCD and pollen development. Emerging evidence suggests the critical role of hypoxia status in specifying anther cell identity, subsequently the triggering signal of ROS as tapetal PCD to nourish microspore for producing mature pollen grains in rice, maize and *Arabidopsis*. During early anther development, the hypoxia status is finely modulated by genetic and metabolic components such as glutaredoxins and TGA factors which are directly or indirectly affected by OsTDL1A-MSP1 signaling pathway.

GRX and TGA counterparts as well as OsTDL1A-MSP1 signaling pathway have been identified to be conserved in different plants species, however, their expression pattern, possible combination of counterparts as well as the genetic function have variation among plants, highlighting the complexity of regulatory network of redox status during evolution. Surprisingly, OsERS1, a glutamyl-tRNA synthetase, is able to maintain proper somatic cell division and organization and limit the over proliferation of male germ cells in rice during early anther development by affecting amino acids hemostasis and redox status regulation. This pathway has not been characterized in other plants. After the anther specification is complete, ROS may trigger tapetal PCD and some transcription factors such as bHLHs, MADS-box proteins may regulate ROS scavengers to maintain redox homeostasis for normal development. As the advance of genomics, genome-editing technologies, more regulators controlling redox status will be elucidated which is critical for both fundamental biology and plant breeding by manipulating male fertility.

## Accession Numbers

The accession numbers for genes mentioned are listed: *AMS* (AT2G16910), *bHLH010* (AT2G31220), *bHLH089* (AT1G06170), *bHLH091* (AT2G31210), *PAN* (AT1G68640), *RBOHE* (AT1G19230), *ROXY1* (AT3G02000), *ROXY2* (AT5G14070), *TGA9* (AT1G08320), *TGA10* (AT5G06839), *FEA4* (Zm00001d037317), *MSCA1* (Zm00001d018802), *MIL1* (Os07g0151100), *DTC1* (Os07g0540366), *EAT1* (Os04g0599300), *MADS3* (Os01g0201700), *MSP1* (Os01g0917500), *OsAP25* (Os03g0186900), *OsAP37* (Os04g0448500), *OsCP1* (Os04g0670500), *OsERS1* (Os10g0369000), *OsGRX_I1* (Os01g0667900), *OsMT2b* (Os05g0111300), *OsMT-I-4b* (Os12g0571100), *OsTDL1A* (Os12g0152500), *OsTGA1* (Os05g0443900), *OsTGA10* (Os09g0489500), *OsTGA11* (Os11g0152700), *OsTGA12* (Os12g0152900), *TIP2* (Os01g0293100), *TDR* (Os02g0120500).

## Data Availability

Not applicable.

## Abbreviations

aaRS: Aminoacyl-tRNA synthetases
Ar: Archesporial cell
DAB: 3,3′-diaminobenzidine
Dds: Dyads
dML: Degenerated middle layer
dT: Degenerated tapetum
E: Epidermis
En: Endothecium
GRX: Glutaredoxin
L2-d: L2-derived cell
LRR-RLK: Leucine-rich repeat receptor-like kinases
ML: Middle layer
MMC: Microspore mother cell
Msp: Microspore
MT: Metallothionein
PCD: Programmed cell death
PPC: Primary parietal cell
Sp: Sporogenous cell
SPC: Secondary parietal cell
T: Tapetum
Tds: Tetrads

## References

[CR1] Apel K, Hirt H (2004). REACTIVE OXYGEN SPECIES: metabolism, oxidative stress, and signal transduction. Annu Rev Plant Biol.

[CR2] Ariizumi T, Toriyama K (2011). Genetic regulation of sporopollenin synthesis and pollen exine development. Annu Rev Plant Biol.

[CR3] Berg M, Rogers R, Muralla R, Meinke D (2005). Requirement of aminoacyl- tRNA synthetases for gametogenesis and embryo development in *Arabidopsis*. Plant J.

[CR4] Cai W, Zhang DB (2018). The role of receptor-like kinases in regulating plant male reproduction. Plant Reprod.

[CR5] Chen ZS, Liu XF, Wang DH, Chen R, Zhang X, Xu ZH, Bai SN (2017). Transcription factor OsTGA10 is a target of the MADS protein OsMADS8 and is required for tapetum development. Plant Physiol.

[CR6] Fu Z, Yu J, Cheng X, Zong X, Xu J, Chen M, Li Z, Zhang D, Liang W (2014). The rice basic Helix-loop-Helix transcription factor TDR INTERACTING PROTEIN2 is a central switch in early anther development. Plant Cell.

[CR7] Gadjev I, Stone JM, Gechev TS (2008). Programmed cell death in plants: new insights into redox regulation and the role of hydrogen peroxide. Int Rev Cell Mol Biol.

[CR8] Gechev TS, Hille J (2005). Hydrogen peroxide as a signal controlling plant programmed cell death. J Cell Biol.

[CR9] Gechev TS, Van Breusegem F, Stone JM, Denev I, Laloi C (2006). Reactive oxygen species as signals that modulate plant stress responses and programmed cell death. BioEssays.

[CR10] Hong L, Tang D, Shen Y, Hu Q, Wang K, Li M, Lu T, Cheng Z (2012). MIL2 (MICROSPORELESS2) regulates early cell differentiation in the rice anther. New Phytol.

[CR11] Hong L, Tang D, Zhu K, Wang K, Li M, Cheng Z (2012). Somatic and reproductive cell development in rice anther is regulated by a putative glutaredoxin. Plant Cell.

[CR12] Hu L, Liang W, Yin C, Cui X, Zong J, Wang X, Hu J, Zhang D (2011). Rice MADS3 regulates ROS homeostasis during late anther development. Plant Cell.

[CR13] Ji C, Li H, Chen L, Min X, Wang F, Chen Y, Liu YG (2013). A novel rice bHLH transcription factor, DTD, acts coordinately with TDR in controlling tapetum function and pollen development. Mol Plant.

[CR14] Kelliher T, Walbot V (2012). Hypoxia triggers meiotic fate acquisition in maize. Science.

[CR15] Ko SS, Li MJ, Sunben KM, Ho YC, Lin YJ, Chuang MH, Hsing HX, Lien YC, Yang HT, Chang HC (2014). The bHLH142 transcription factor coordinates with TDR1 to modulate the expression of EAT1 and regulate pollen development in rice. Plant Cell.

[CR16] Lam E, Pozo OD, Pontier D (2001) Involvement of ROS and caspase-like proteases during cell death induction by plant pathogens. In: Signal transduction in plants. Springer US

[CR17] Lee S, Jung KH, An G, Chung YY (2004). Isolation and characterization of a rice cysteine protease gene, OsCP1, using T-DNA gene-trap system. Plant Mol Biol.

[CR18] Lemaire SD (2004). The glutaredoxin family in oxygenic photosynthetic organisms. Photosynth Res.

[CR19] Li N, Zhang D, Liu H, Yin C, Li X, Liang W, Yuan Z, Xu B, Chu H, Wang J, Wen T, Huang H, Luo D, Ma H, Zhang D (2006). The rice tapetum degeneration retardation gene is required for tapetum degradation and anther development. Plant Cell.

[CR20] Li S., Lauri A., Ziemann M., Busch A., Bhave M., Zachgo S. (2009). Nuclear Activity of ROXY1, a Glutaredoxin Interacting with TGA Factors, Is Required for Petal Development in Arabidopsis thaliana. THE PLANT CELL ONLINE.

[CR21] Miller G, Mittler R (2006). Could heat shock transcription factors function as hydrogen peroxide sensors in plants?. Ann Bot.

[CR22] Mittler R, Vanderauwera S, Gollery M, Van Breusegem F (2004). Reactive oxygen gene network of plants. Trends Plant Sci.

[CR23] Murmu J, Bush MJ, DeLong C, Li S, Xu M, Khan M, Malcolmson C, Fobert PR, Zachgo S, Hepworth SR (2010). *Arabidopsis* basic leucine-zipper transcription factors TGA9 and TGA10 interact with floral glutaredoxins ROXY1 and ROXY2 and are redundantly required for anther development. Plant Physiol.

[CR24] Niu N, Liang W, Yang X, Jin W, Wilson ZA, Hu J, Zhang D (2013). EAT1 promotes tapetal cell death by regulating aspartic proteases during male reproductive development in rice. Nat Commun.

[CR25] Nonomura KI, Miyoshi K, Eiguchi M, Suzuki T, Miyao A, Hirochika H, Kurata N (2003). The MSP1 gene is necessary to restrict the number of cells entering into male and female sporogenesis and to initiate anther wall formation in rice. Plant Cell.

[CR26] Overmyer K, Brosche ´ M, Kangasja ¨rvi J (2003). Reactive oxygen species and hormonal control of cell death. Trends Plant Sci.

[CR27] Pautler M, Eveland AL, LaRue T, Yang F, Weeks R, Lunde C, Je BI, Meeley R, Komatsu M, Vollbrecht E, Sakai H, Jackson D (2015). FASCIATED EAR4 encodes a bZIP transcription factor that regulates shoot meristem size in maize. Plant Cell.

[CR28] Rouhier N, Gelhaye E, Jacquot JP (2004). Plant glutaredoxins: still mysterious reducing systems. Cell Mol Life Sci.

[CR29] Running M, Meyerowitz (1996) Mutations in the PERIANTHIA gene of *Arabidopsis* specifically alter floral organ number and initiation pattern. Development 122:1261–1269 10.1242/dev.122.4.1261 8620853

[CR30] Scherz-Shouval R, Shvets E, Fass E, Shorer H, Gil L, Elazar Z (2007). Reactive oxygen species are essential for autophagy and specifically regulate the activity of Atg4. EMBO J.

[CR31] Sheridan WF, Avalkina NA, Shamrov II, Batygina TB, Golubovskaya IN (1996). The *mac1* gene: controlling the commitment to the meiotic pathway in maize. Genetics.

[CR32] Sheridan WF, Golubeva EA, Abrhamova LI, Golubovskaya IN (1999). The *mac1* mutation alters the developmental fate of the hypodermal cells and their cellular progeny in the maize anther. Genetics.

[CR33] Shi J, Cui M, Yang L, Kim YJ, Zhang D (2015). Genetic and biochemical mechanisms of pollen wall development. Trends Plant Sci.

[CR34] Wong HL, Sakamoto T, Kawasaki T, Umemura K, Shimamoto K (2004). Down-regulation of metallothionein, a reactive oxygen scavenger, by the small GTPase OsRac1 in rice. Plant Physiol.

[CR35] Xie T, Wang Z, Li S, Zhang Y (2014). Spatiotemporal production of reactive oxygen species by NADPH oxidase is critical for tapetal programmed cell death and pollen development in *Arabidopsis*. Plant Cell.

[CR36] Xing S, Zachgo S (2010). ROXY1 and ROXY2, two *Arabidopsis* glutaredoxin genes, are required for anther development. Plant J.

[CR37] Xu J, Ding Z, Schreiber L, Wilson ZA, Zhang D (2014). ABORTED MICROSPORES acts as a master regulator of pollen wall formation in *Arabidopsis*. Plant Cell.

[CR38] Xu J, Yang C, Yuan Z, Zhang D, Gondwe MY, Ding Z, Liang W, Zhang D, Wilson ZA (2010). The ABORTED MICROSPORES regulatory network is required for postmeiotic male reproductive development in *Arabidopsis thaliana*. Plant Cell.

[CR39] Yamakawa H, Hakata M (2010). Atlas of rice grain filling-related metabolism under high temperature: joint analysis of metabolome and transcriptome demonstrated inhibition of starch accumulation and induction of amino acid accumulation. Plant Cell Physiol.

[CR40] Yang F, Bui HT, Pautler M, Llaca V, Johnston R, Lee BH, Kolbe A, Sakai H, Jackson D (2015). A maize glutaredoxin gene, abphyl2, regulates shoot meristem size and phyllotaxy. Plant Cell.

[CR41] Yang L, Qian X, Chen M, Fei Q, Meyers BC, Liang W, Zhang D (2016). Regulatory role of a receptor-like kinase in specifying anther cell identity. Plant Physiol.

[CR42] Yang X, Li G, Tian Y, Song Y, Liang W, Zhang D (2018). A rice glutamyl-tRNA synthetase modulates early anther cell division and patterning. Plant Physiol.

[CR43] Yi J, Moon S, Lee YS, Zhu L, Liang W, Zhang D, Jung KH, An G (2016). Defective tapetum cell death 1 (DTC1) regulates ROS levels by binding to metallothionein during tapetum degeneration. Plant Physiol.

[CR44] Zhang D, Luo X, Zhu L (2011). Cytological analysis and genetic control of rice anther development. J Genet Genomics.

[CR45] Zhang D, Yang L (2014). Specification of tapetum and microsporocyte cells within the anther. Cur Opin Plant Biol.

[CR46] Zhao GC, Shi JX, Liang WQ, Zhang DB (2016). ATP binding cassette G transporters and plant male reproduction. Plant Signal Behav.

[CR47] Zhu E, You C, Wang S, Cui J, Niu B, Wang Y, Qi J, Ma H, Chang F (2015). The DYT1-interacting proteins bHLH010, bHLH089 and bHLH091 are redundantly required for *Arabidopsis* anther development and transcriptome. Plant J.

